# Study on the Tensile Properties and Waterproofing Mechanism of Bamboo Fibers Treated by Different Methods

**DOI:** 10.3390/polym17233146

**Published:** 2025-11-26

**Authors:** Chuncheng Sun, Haiying Cao, Enhua Zhang, Jiefeng Liu

**Affiliations:** Hebei Key Laboratory of Green Construction and Intelligent Maintenance for Civil Engineering, Hebei Province Engineering Research Center for Harmless Synergistic Treatment and Recycling of Municipal Solid Waste, School of Civil Engineering and Mechanics, Yanshan University, Qinhuangdao 066004, China; 15690261176@163.com (C.S.); 18732564021@163.com (E.Z.); liujiefeng@ysu.edu.cn (J.L.)

**Keywords:** bamboo fiber, sodium silicate solution treatment, water immersion test, tensile strength, young’s modulus, waterproofing mechanism

## Abstract

Bamboo fibers have received significant attention due to their biodegradability and environmental benefits. However, their inherent hydrophilicity causes dramatic degradation in mechanical properties after water absorption. Some methods have been adopted to treat bamboo fiber to address this challenge, e.g., sodium hydroxide (NaOH) solution treatment, vegetable oil treatment, and carboxylated styrene butadiene rubber (XSBR) treatment. In this study, the sodium silicate solution treatment method is proposed. The effects of four treatment methods on bamboo fibers are systematically evaluated using direct tensile tests, scanning electron microscopy (SEM), and Fourier transform infrared spectroscopy (FTIR). The results indicate that all four treatment methods can effectively mitigate the reduction in tensile stress and Young’s modulus of bamboo fibers after water immersion. Sodium silicate solution (modulus = 3.3)-treated bamboo fibers show the smallest reduction in tensile strength (36.8%), while the Young’s modulus of the sodium silicate solution (modulus = 2.3)-treated fibers increased by 4.5%. FTIR analysis shows that four treatment methods lead to reduction in hydrophilic groups in bamboo fiber. For the sodium silicate solution treatment method, a hydrophobic solidified sodium silicate layer forms on the surface of bamboo fibers, which further hinders moisture absorption.

## 1. Introduction

In recent years, the concept of green sustainability has emerged as a key focus in engineering and technology. Among these, natural fibers have garnered considerable attention owing to their biodegradability, environmental friendliness, and high specific strength, making them particularly relevant for construction materials. For example, bamboo fibers [1,2], coir fibers [3,4,5,6], and jute fibers [7,8] have been utilized as reinforcement materials in cement-based composites to improve their mechanical properties. Additionally, sisal fibers [9] and jute fibers [10], combined with enzyme-induced carbonate precipitation (EICP) and microbial-induced carbonate precipitation (MICP) techniques, have shown excellent results in soil stabilization. Surface-treated bamboo fibers have also demonstrated enhanced interfacial properties with asphalt [11].

Bamboo is an exceptionally abundant resource, with global annual production estimated at 50–60 million metric tons, characterized by its short harvesting rotation cycles [12,13,14]. As a representative example of natural fibers [15,16], bamboo fiber’s specific strength is similar to that of glass fibers [17]. In comparison with other natural fibers, e.g., kenaf, cedar, and ramie, bamboo fiber demonstrates superior tensile strength and modulus [18]. Consequently, the engineering applications of bamboo fiber have received more and more attention from researchers. Terai et al. [19] incorporated varying dosages of bamboo fibers into concrete and reported improvements in flexural strength, tensile strength, and compressive strength. Ramaswamy et al. [20] investigated the effects of bamboo fiber inclusion on the deformation behavior of concrete, demonstrating its effectiveness in mitigating shrinkage deformation. Chin et al. [21] demonstrated the potential of bamboo fiber composite plates (BFCP) for enhancing the flexural performance of reinforced concrete (RC) beams. Akinyemi et al. [22] studied the effects of different pre-treatment methods on bamboo fiber cement composite materials and observed that microwave-assisted alkali treatment enhanced mechanical properties. Sanchez-Echeverri et al. [23] analyzed the impact of alkali-treated bamboo fibers on improving the flexural strength of cement-based materials. Huang et al. [24] explored the feasibility of utilizing bamboo fiber and bamboo charcoal as construction fillers in tropical and subtropical regions.

However, the engineering application of bamboo fibers is limited due to their capacity for high-water absorption, with a utilization rate of only approximately 40% [25]. Bamboo fibers can absorb between 100 and 300% of their dry weight in moisture [26,27,28]. It is primarily attributed to the anatomical composition of bamboo, which consists of cell walls of fiber cells, parenchyma cells, and vessel cells [29]. All of which contain numerous hydrophilic groups, particularly free -OH, which confer strong water absorption characteristics to bamboo [29,30]. The significant hydrophilicity of bamboo fibers has several detrimental effects, including structural softening, reductions in both tensile strength and Young’s modulus. When used as a reinforcement material, the high hygroscopicity of bamboo fibers can lead to repeating cycles of swelling and shrinkage, which results in the integrity of the fiber–matrix interface and weakening of the overall adhesion characteristics [28,31,32].

To prevent degradation in the mechanical properties of bamboo fibers from moisture absorption during engineering applications, many studies have focused on modification research on bamboo fibers. Currently, treatment methods for bamboo fibers are generally classified into three categories, i.e., chemical treatment, hygrothermal treatment, and physicochemical treatment. Among these, chemical treatments, such as alkali treatment [17,33] and silane treatment [34], are commonly used. For example, Geremew et al. [35] found that NaOH solution treatment significantly reduces the water absorption of bamboo fibers. Hygrothermal treatments are represented by steam treatment [29,36] and vegetable oil treatment [28]. Bui et al. [28] demonstrated that oil treatment effectively reduces bamboo’s sensitivity to moisture, thereby improving its durability. Physicochemical treatment mainly includes carboxylated styrene butadiene rubber (XSBR) treatment [37]. Ferreira et al. [37] found that XSBR treatment can generally reduce the water absorption rate of various natural fibers. These treatment methods essentially modify the structural organization of the fiber matrix, a principle that has also been observed in the modification of other types of fibers. For example, Makarov et al. [38] demonstrated that various treatments can alter the supramolecular structure of cellulose fibers, thereby improving their mechanical properties. However, each of the above methods has its limitations. Chemical treatment can reduce water absorption by removing hemicellulose by increasing the alkali concentration, but it often results in a significant decrement in the tensile strength of bamboo fibers [33]. Hygrothermal treatment utilizes steam or vegetable oil as processing media to degrade cellulose and hemicellulose under high temperatures. However, this method is complex, of long duration, and high cost. Physical–chemical treatment, represented by XSBR, can suppress water absorption and maintain a certain tensile strength of bamboo fibers, but it poses potential risks to the ecological environment due to high degradation resistance, unpleasant odor, and heavy metal content of XSBR. Therefore, finding an economical, environmentally friendly, and efficient bamboo fiber treatment method has emerged as a key research priority in this field.

Sodium silicate solution, as a low-cost, non-toxic, colorless, odorless, environmentally friendly material, has shown potential in waterproofing treatments. For example, Song et al. [39] found that sodium silicate can effectively improve the compactness and water impermeability of concrete; Li et al. [40] demonstrated that the water absorption of fir wood treated by sodium silicate was significantly reduced. Based on the above, this study proposes the use of sodium silicate solution for the treatment of bamboo fibers.

In this study, the performance of sodium silicate solution treatment is compared with three traditional methods (NaOH solution, vegetable oil, and XSBR). Direct tensile tests are conducted on raw and treated bamboo fibers before and after water immersion, and the corresponding stress–strain curves are analyzed. In addition, Fourier transform infrared spectroscopy (FTIR) and scanning electron microscopy (SEM) are used to characterize the chemical composition and microstructure of the bamboo fibers before and after treatment, further revealing the impact of different treatment mechanisms on their properties.

## 2. Method

### 2.1. Material Properties and Selection Criteria for Natural Bamboo Fibers

The bamboo fibers used in this study are sourced from a commercial supplier in Sichuan Province, China, and are from the same batch of production. These fibers are derived from *Phyllostachys pubescens* (Moso bamboo) and are characterized by a high density and low content of sugar, starches, and proteins. The tensile strength ranges from 550 MPa to 650 MPa, and Young’s modulus is about 20 GPa; the degree of polymerization of cellulose is in the range of 1200–1600. The fundamental chemical composition of bamboo fibers is summarized in Table 1.

To ensure statistical reliability, a total of 120 bamboo fiber specimens are tested, as outlined in Table 2. To minimize experimental error, all fibers in the test batch are standardized to a diameter of 0.3 ± 0.05 mm and lengths of 45 ± 0.1 mm.

### 2.2. Materials and Treatments

(a) Sodium hydroxide (NaOH) solution treatment

The treatment utilized a 5% NaOH solution (pH = 13) at a laboratory ambient temperature of 26 °C, with a fiber-to-solution mass ratio of 1:100. The specific procedure, adapted from [17], involved immersing bamboo fibers in NaOH solution with intermittent stirring every 30 min to maintain solution homogeneity. After 2 h of treatment, individual bamboo fibers are thoroughly rinsed with deionized water to remove residual NaOH and subsequently oven-dried at 80 °C for 6 h to eliminate excess moisture.

(b) Vegetable oil treatment

Vegetable oil treatment is conducted using sunflower oil at a laboratory ambient temperature of 26 °C [28]. Natural bamboo fibers are first oven-heated at 180 °C for 1 h to degrade sugars, starches, and proteins within the fibers [41,42], and then cooled in sunflower oil at 26 °C. It is worth noting that this heating process (temperature exceeding 160 °C) can cause a significant decrease in the mechanical properties of bamboo fibers [29]. The fibers are removed and subsequently air-stored for two weeks under ambient laboratory conditions.

(c) XSBR treatment

The XSBR treatment employed an emulsion with a 48% solid content and a pH = 8 (weakly alkaline), conducted at a laboratory ambient temperature of 26 °C. Following the procedure described in [37], natural bamboo fibers are immersed in the XSBR emulsion for 50 min, then retrieved and air-dried at room temperature for 24 h. Prior to FTIR spectroscopic analysis, the treated bamboo fibers are thoroughly washed to remove any residual XSBR from the surface.

(d) Sodium silicate solution treatment

Bamboo fibers are treated by sodium silicate solutions of two different moduli: 2.3 and 3.3. The modulus in this context refers to the SiO_2_/Na_2_O mass ratio, which is a dimensionless value that indicates the relative proportions of silicon dioxide (SiO_2_) and sodium oxide (Na_2_O) in the sodium silicate solution. The modulus 2.3 solution contained 30 wt% SiO_2_, 13.5 wt% Na_2_O, with a Baumé degree of 50°Bé, solid content of 43.5%, and pH = 12. The modulus 3.3 solution contained 26.98 wt% SiO_2_, 8.53 wt% Na_2_O, with a Baumé degree of 38.5°Bé, solid content 35.5%, and pH = 10. All treatments are conducted at a laboratory ambient temperature of 26 °C. Bamboo fibers are fully immersed in the respective sodium silicate solution for 3 h, then retrieved and oven-dried at 130 °C for 1 h to promote curing reactions of the adhered silicate. Sodium silicate solution typically undergoes condensation of Si-OH groups within the temperature range of 120–300 °C, forming water-resistant Si-O-Si networks. However, heating above 160 °C significantly degrades the mechanical properties of bamboo fibers [29]. Therefore, a curing temperature of 130 °C is selected as representing an optimal compromise sufficient to promote the formation of a hydrophobic silicate network while preserving the integrity of the bamboo fibers.

## 3. Testing

### 3.1. Water Immersion Softening Test

**Figure 1:**
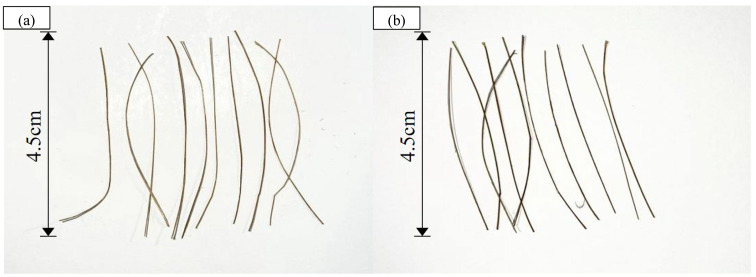
Comparison of bamboo fibers before and after water immersion. (**a**) before water immersion; (**b**) after water immersion.

To examine the evolution of mechanical properties following water absorption, bamboo fibers are immersed in water for 5 days to achieve full saturation. After water immersion, the fibers are retrieved, and surface moisture is carefully blotted prior to the tensile test. Figure 1 presents a comparison of the bamboo fibers before and after water immersion, with each bamboo fiber approximately 4.5 cm in length.

### 3.2. Direct Tensile Test

The tensile test is conducted using an INSTRON universal test machine, with a measurement accuracy of 0.5%. In accordance with ASTM C1557 [43], tensile tests are conducted under displacement control at a loading rate of 0.5 mm/min. For each of the five fiber types (raw and four treated variants), ten specimens are tested before and after water immersion. After excluding maximum/minimum values as well as defective samples (e.g., those exhibiting visible bending damage), six valid test results are retained for analysis. Prior to testing, a single-fiber fixation device is developed based on methods described in references [44,45]. This device is designed to prevent direct gripper contact with bamboo fibers, thereby avoiding stress concentration while ensuring secure and eccentricity-free alignment. The device consists of a 4 cm × 4 cm transparent paper frame, which is made by cutting out the center of a piece of transparent paper. The bamboo fiber is fixed to both sides of the transparent paper frame using glue, and each fiber has a free length of 3 cm. Shortly before the test, the paper frame will be cut along the cutting line, preventing simultaneous loading of the paper and fiber. With this preparation, the bamboo fiber at the clamping area is strengthened by the glue, allowing the grippers to make contact only with the adhesive and the paper when clamping both sides of the transparent paper frame.

The tests initially produce load–displacement curves, which are converted into stress–strain curves using Equations (1) and (2):

(1) σ=FA

where *F* is the tensile load (*N*) and *A* is the cross-sectional area of the bamboo fiber (mm^2^).

(2) ε=Δll

where ∆*l* is the displacement increment of the bamboo fiber (mm) and *l* is the original gauge length of the bamboo fiber (30 mm).

### 3.3. Scanning Electron Microscopy (SEM) Analysis

Microstructural characterization is performed using a Hitachi S-3400N scanning electron microscope. Prior to imaging, all samples are sputter-coated with gold to enhance conductivity. Observations are conducted at an accelerating voltage of 15 kV and a tilt angle of 0°. SEM analyses focus on evaluating the surface morphology, tensile fracture surfaces, and mechanically cut surfaces of all five types of bamboo fibers.

### 3.4. Fourier Transform Infrared Spectroscopy (FTIR) Analysis

The FTIR spectra are collected using a Nicolet iN10 spectrometer (Thermo Scientific, Waltham, MA, USA) over a range of 4000–500 cm^−1^, with a resolution of 4 cm^−1^ and 16 scans per sample. Prior to analysis, KBr pellets are prepared by thoroughly mixing finely ground bamboo fiber powder with KBr at a weight ratio of 100:1 (KBr:fiber), followed by vacuum compression.

## 4. Results and Analysis

### 4.1. Tensile Strength Analysis

**Figure 2:**
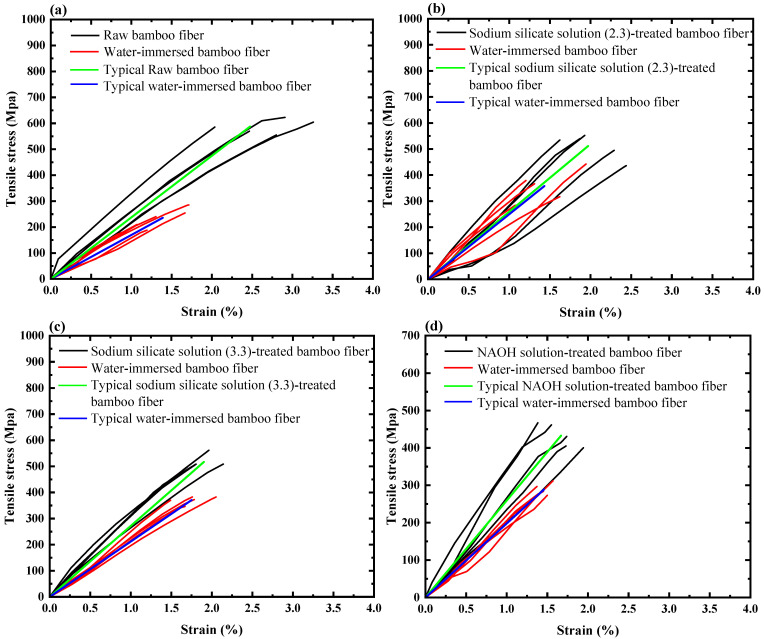

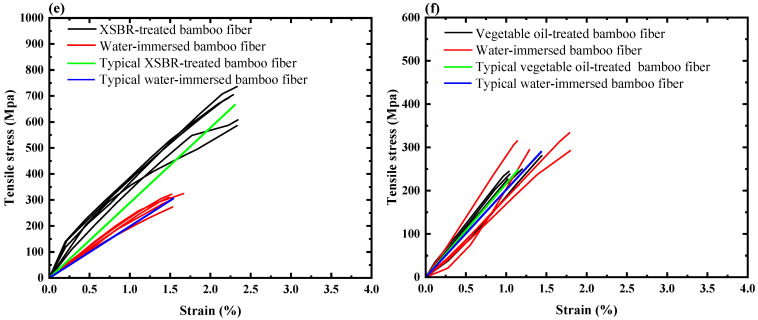
Stress–strain curves of five types of bamboo fiber before and after water immersion. (**a**) raw bamboo fibers; (**b**) sodium silicate solution (modulus = 2.3)-treated bamboo fibers; (**c**) sodium silicate solution (modulus = 3.3)-treated bamboo fibers; (**d**) NaOH solution-treated bamboo fibers; (**e**) XSBR-treated bamboo fibers; and (**f**) vegetable oil-treated bamboo fibers.

Figure 2 presents the relationship between tensile strength and axial strain of bamboo fibers under various treatment methods. For every treatment method, two groups of stress–strain curves are shown, i.e., the before-water-immersion group (with representative curves) and the after-water-immersion group (with representative curves). It can be observed that tensile stress increases almost linearly with an increase in strain, and the fracture strain ranges from 1% to 3.5%, except for the bamboo fibers treated with vegetable oil. The slopes of the stress–strain curve of all water-immersed bamboo fibers exhibit a decreasing trend compared to that of bamboo fibers before water immersion.

The properties of raw bamboo fibers and four types of treated bamboo fibers before water immersion are presented in Table 3: NaOH solution-treated bamboo fibers exhibited a 26.2% reduction in tensile strength but a 6.6% increment in Young’s modulus compared with raw fibers. Vegetable oil-treated bamboo fibers exhibited obvious decreases in both tensile strength (57.8%) and Young’s modulus (9.1%). In contrast, XSBR-treated bamboo fibers exhibited increases in tensile strength and Young’s modulus by 13.3% and 21.7%, respectively. In addition, bamboo fibers treated with sodium silicate solution (modulus = 2.3) displayed a 12.9% decrement in tensile strength alongside a 9.3% increment in Young’s modulus. Sodium silicate solution-treated (modulus = 3.3) bamboo fibers showed an 11.9% reduction in tensile strength and a 14.4% increment in Young’s modulus.

Both NaOH solution and sodium silicate solution are alkaline solutions. Upon full contact with bamboo fibers, they facilitate the degradation of hemicellulose and lignin, leading to reductions in fiber diameter, lumen size, and cross-sectional area, as well as cracks in cell walls [33]. These changes result in a decrease in the tensile strength of bamboo fibers. The pH of NaOH solution is higher than that of sodium silicate solution, which leads to more severe degradation of bamboo fibers and consequently yields a lower tensile strength than that of sodium silicate solution. Additionally, sodium silicate solution (modulus = 2.3) exhibits a higher pH than sodium silicate solution (modulus = 3.3), which also results in a higher reduction in the tensile strength of the sodium silicate solution (modulus = 2.3)-treated bamboo fibers. Moreover, the tensile strengths of bamboo fibers treated by alkaline solutions follow the order: sodium silicate solution (modulus = 3.3) > sodium silicate solution (modulus = 2.3) > NaOH solution.

Alkaline solutions partially ionize the -OH groups of cellulose of bamboo fiber [46], leading to a rearrangement of the molecular chains. The rearrangement promotes chains to form tighter bonds; thereby, the ability to resist deformation is enhanced, and Young’s modulus is increased. However, as pH increases, the cellulose lattice structure becomes increasingly disrupted, which reduces the improvement in Young’s modulus. Consequently, the order of Young’s modulus for bamboo fibers treated with alkaline solutions is as follows: sodium silicate solution (modulus = 3.3) > sodium silicate solution (modulus = 2.3) > NaOH solution.

Bamboo fibers treated with vegetable oil show a reduction in tensile strength and Young’s modulus compared to those of raw bamboo fiber. It can be explained as follows: First, during the high-temperature baking stage at 180 °C, excessive moisture evaporation leads to structural shrinkage and the development of local stress, and the disruption of partial hydrogen-bonding structures weakens the intermolecular forces. Second, the elevated temperature also induces the degradation of cellulose, hemicellulose, and lignin [28,29]. These changes are reflected macroscopically as a decrease in both tensile strength and Young’s modulus.

In contrast to the alkaline solutions and vegetable oil treatments, bamboo fibers treated with XSBR exhibited improved tensile strength. The improvement can be attributed to several factors: (1) XSBR polymerization on the fiber microstructure, mainly at the surface, which allows interlocking between the fiber cell walls. The porosity of fiber is partially decreased and stiffens its structure [37]; (2) as an elastomeric material with inherent tensile strength, during the tensile test, the XSBR and bamboo fibers jointly bore the external load [37].

After water immersion, the tensile strength and Young’s modulus of all five types of bamboo fibers exhibited a reduction trend. As shown in Table 3, compared to the initial properties of raw bamboo fibers before water immersion, the tensile strength and Young’s modulus of raw bamboo fibers decreased by 60.0% and 28.6%, respectively. For NaOH solution-treated bamboo fibers, they decreased by 50.9% and 17.0%, respectively. For XSBR-treated bamboo fibers, they decreased by 48.0% and 16.4%, respectively. For sodium silicate solution (modulus = 3.3)-treated bamboo fibers, they decreased by 36.8% and 10.7%, respectively. For sodium silicate solution (modulus = 2.3)-treated bamboo fibers, the tensile strength decreased by 39.0%, while the Young’s modulus increased by 4.5%. Clearly, bamboo fibers treated by sodium silicate solution (modulus = 3.3) exhibited both the lowest tensile strength reduction rate and the highest tensile strength after water immersion, while the bamboo fibers treated by sodium silicate solution (modulus = 2.3) had the highest Young’s modulus after water immersion.

The mechanical properties of all five types of bamboo fibers decreased after water immersion. The reduction can be attributed to the high-water absorption of bamboo fibers, which leads to swelling. The resulting structural expansion induces internal softening and damage [47,48]. And then, degradation in mechanical performance is observed.

Table 4 presents a comparative assessment of various bamboo fiber treatment methods, evaluating their environmental friendliness, costs, and the resulting mechanical performance of treated bamboo fibers. Although XSBR-treated bamboo fibers exhibit the highest tensile strength and Young’s modulus before water immersion, XSBR is associated with high cost, poor biodegradability, an irritating odor, and the presence of heavy metals and other harmful substances, all of which pose potential environmental risks [49]. NaOH solution-treated bamboo fibers demonstrate relatively low tensile strength both before and after water immersion. Among all treatment methods, vegetable oil-treated bamboo fibers exhibited the lowest tensile strength under both dry and wet conditions.

Therefore, considering costs, environmental friendliness, and mechanical performance, sodium silicate solution demonstrates clear advantages among the five treatment methods for bamboo fibers. It is low-cost, environmentally benign, non-toxic, safe, and effectively preserves the mechanical properties of bamboo fibers, thus representing a green treatment method with engineering application prospects.

### 4.2. Chemical Composition Analysis

**Figure 3:**
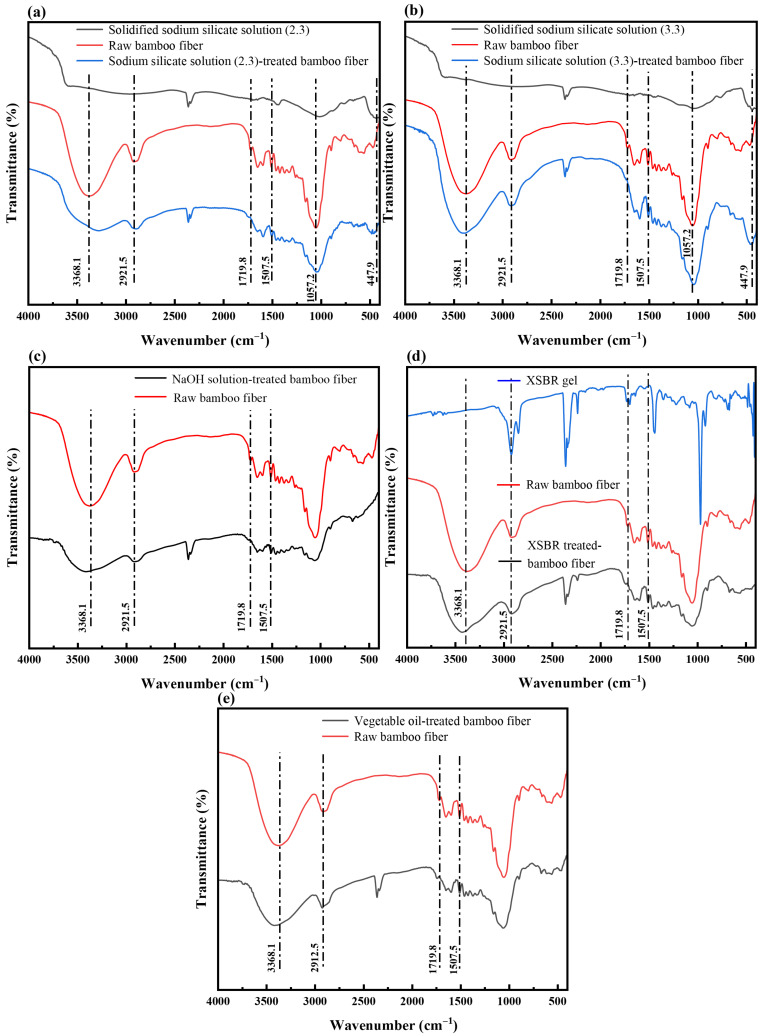
FTIR spectroscopy of raw bamboo fibers and four types of treated bamboo fibers. (**a**) sodium silicate solution (modulus = 2.3)-treated bamboo fibers; (**b**) sodium silicate solution (modulus = 3.3)-treated bamboo fibers; (**c**) NaOH solution-treated bamboo fibers; (**d**) XSBR-treated bamboo fibers; and (**e**) vegetable oil-treated bamboo fibers.

The four treatment methods significantly modify the chemical composition of bamboo fibers before water immersion. The -OH groups present in cellulose, hemicellulose, and lignin are the key determinants of water absorption of bamboo fiber. FTIR spectroscopy is employed to analyze changes in the structural components and their -OH groups. As illustrated in Figure 3, the absorption peaks observed in the range of 2990–3660 cm^−1^ correspond to the -OH stretching vibrations of cellulose, hemicellulose, and lignin [50]. The peak at 2921.5 cm^−1^ is a characteristic adsorption shared by both cellulose and lignin [34,50].

Peaks in the range of 1694–1737 cm^−1^ are attributed to the C=O stretching vibrations of hemicellulose [33,50,51]. Additionally, peaks at 1510–1513 cm^−1^ are indicative of the aromatic ring skeleton vibration characteristic of lignin [51,52]. Notably, the peaks at 1057.2 cm^−1^ and 447.9 cm^−1^ in Figure 3a,b correspond to the Si-O stretching vibration. The formation of Si-O bonds could be explained by Equation (3): sodium silicate solution, which contains abundant Si-OH groups and undergoes catalyzed condensation upon heating. At 130 °C, residual water molecules facilitate silanol condensation, resulting in dehydration and the formation of Si-OH groups and Si-O-Si bonds. This reaction leads to the development of a hydrophobic, three-dimensional crosslinked network. The observed peaks confirm the formation of a hydrophobic layer on the bamboo fiber surface after sodium silicate solution treatment.

As observed in Figure 3a,c, the peak intensity at 3368.1 cm^−1^ decreases significantly. This reduction is primarily attributed to the strong alkalinity of sodium silicate solution (modulus = 2.3) and NaOH solution, which disrupts the -OH groups in cellulose, hemicellulose, and lignin. In contrast, Figure 3b,d exhibit minor changes in the intensity at 3368.1 cm^−1^, which suggests that the weaker alkalinity of sodium silicate solution (modulus = 3.3) and XSBR have minimal impact on -OH groups. Additionally, the peak intensities at 2921.5 cm^−1^ and 1512.5 cm^−1^ in Figure 3b,d remain largely unchanged, which indicates that sodium silicate solution (modulus = 3.3) and XSBR induce minimum degradations of cellulose and lignin. In all spectra (Figure 3a–e), the peak at 1719.8 cm^−1^ nearly disappears, suggesting substantial degradation of hemicellulose in treated bamboo fibers. Notably, in Figure 3a,b, the absorption peak at 1057.2 cm^−1^ exhibits a rightward shift, which confirms the formation of Si-O bonds [53]. In Figure 3b, a dramatic increase in intensity is observed at 447.9 cm^−1^, which provides further evidence that more Si-O bonds are generated in sodium silicate solution (modulus = 3.3) during thermal curing, resulting in a denser Si-O-Si hydrophobic network. Compared with raw bamboo fibers, Figure 3e displays significantly reduced peak intensities at 3368.1 cm^−1^, 2921.5 cm^−1^, and 1512.5 cm^−1^, along with the disappearance of the 1719.8 cm^−1^ peak. These changes are primarily attributed to the thermal degradation of cellulose, hemicellulose, and lignin in the bamboo fibers at the elevated temperature of 180 °C.

In addition, among all treatments, -OH groups in bamboo fibers treated with sodium silicate solution (modulus = 3.3) do not show a significant decrease; thus, in theory, this treatment has a limited influence on reducing water absorption. Logically, compared to other treated methods, this would result in higher reduction rates on tensile strength and Young’s modulus of bamboo fibers after water immersion. However, this expectation contradicts the actual tensile test results. So, it is important to clarify the mechanism of the paradoxical experimental observation.Na_2_O·nSiO_2_ + (2n + 1)H_2_O → 2NaOH + nSi(OH)_4_ 
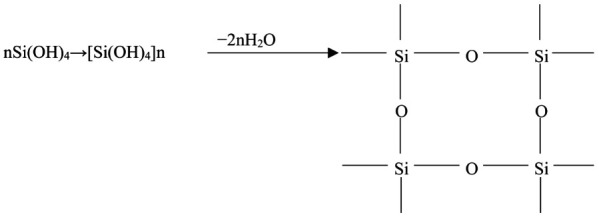
(3)

### 4.3. Microstructural Characterization

#### 4.3.1. Surface Morphological Characteristics of Bamboo Fibers

**Figure 4:**
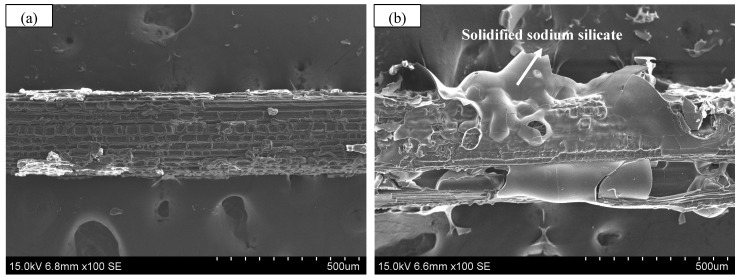

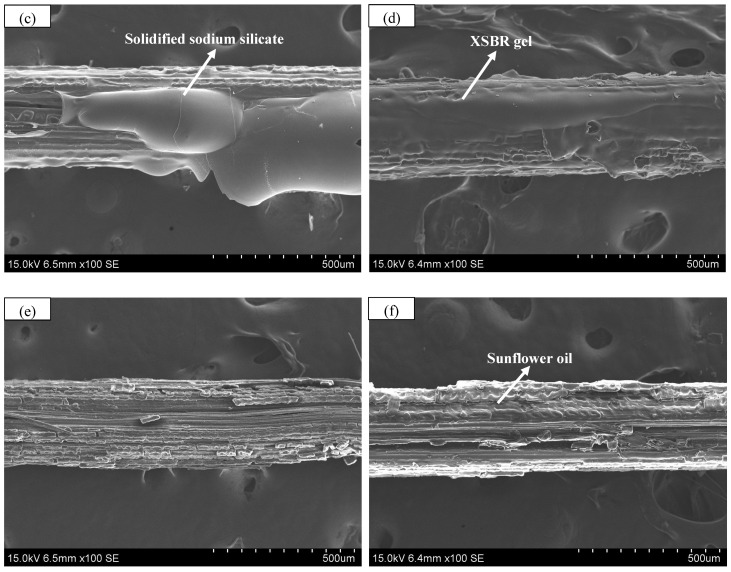
Surface characteristics of five types of bamboo fibers. (**a**) raw bamboo fibers; (**b**) sodium silicate solution (modulus = 2.3)-treated bamboo fibers; (**c**) sodium silicate solution (modulus = 3.3)-treated bamboo fibers; (**d**) XSBR-treated bamboo fibers; (**e**) NaOH solution-treated bamboo fibers, and (**f**) vegetable oil-treated bamboo fibers.

Figure 4 exhibits the surface characteristics of five types of bamboo fibers. As shown in Figure 4a, the surface of raw bamboo fibers is characterized by irregular parallel grooves and striations along the fiber axis. These grooves have different depths, which increases the specific surface area, and the adsorption capacity and interfacial bonding potential of the bamboo fibers are enhanced. The characteristic facilitates the formation of robust composite layer structures between the treatment materials and bamboo fiber surfaces. As shown in Figure 4b,c, the sodium silicate solution forms a solidified layer of measurable thickness on the surface of the bamboo fibers after heat treatment. Simultaneously, a degradation reaction occurs between the bamboo fiber surface and the sodium silicate, resulting in a reduction in hydroxyl (-OH) content (as concluded in Section 4.2). The aforementioned process reduces hydrophilic groups on the bamboo fiber surface through chemical modification, and together with the physical barrier effect of the solidified layer, synergistically enhances the water resistance of the bamboo fibers. Consistent with the observations in Figure 4b,c, Figure 4d shows that XSBR forms a gel-encapsulating layer on the surface of the bamboo fibers. The degradation reaction between XSBR and bamboo fibers similarly reduces the -OH content (as discussed in Section 4.2). As shown in Figure 4e, NaOH solution-treated bamboo fibers exhibit rougher surfaces compared to those in Figure 4a. This results from the partial removal of hemicellulose and lignin due to alkaline degradation [54], with some regions exhibiting more extensive degradation, exposing finer microfibrils, and presenting a “brush-like” surface morphology [55]. Compared to the sodium silicate solution or XSBR treatments, the formation of hydrophobic membrane structures (e.g., solidified layers, gel layers) on the bamboo fiber surface is not found in the NaOH solution treatment. Figure 4f reveals that bamboo fibers are coated with an oil film that provides hydrophobicity, and the high temperature treatment promotes bamboo fiber degradation that reduces the -OH content.

#### 4.3.2. Morphological Characteristics of Mechanically Cut and Tensile-Fractured Surfaces

**Figure 5:**
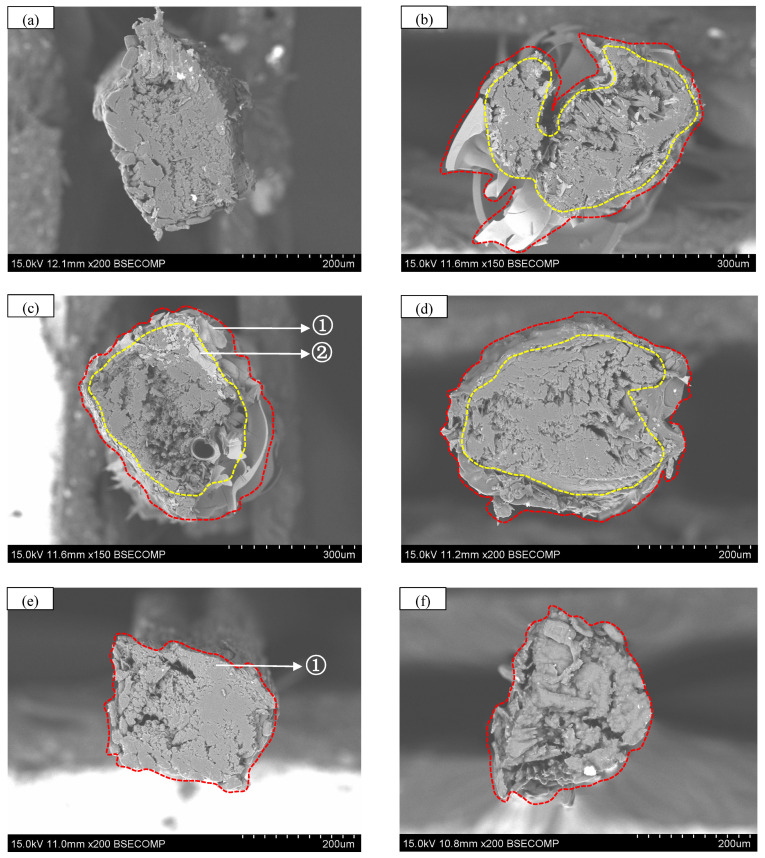
Mechanically cut surfaces of five types of bamboo fibers. (**a**) raw bamboo fibers; (**b**) sodium silicate solution (modulus = 2.3)-treated bamboo fibers; (**c**) sodium silicate solution (modulus = 3.3)-treated bamboo fibers; (**d**) XSBR-treated bamboo fibers; (**e**) NaOH solution-treated bamboo fibers, and (**f**) vegetable oil-treated bamboo fibers.

Figure 5 exhibits the mechanically cut surface of five types of bamboo fibers. Different from the mechanically cut surface of the raw bamboo fiber (Figure 5a), the mechanically cut surfaces of the bamboo fibers treated with sodium silicate solution in Figure 5b,c consist of two parts: the solidified sodium silicate layer and the bamboo fiber itself. It can be observed that the solidified sodium silicate product did not penetrate into the interior of the bamboo fiber. The mechanically cut surface of the bamboo fiber in Figure 5d is composed of two parts: the XSBR gel layer and the bamboo fiber itself. Similarly, no XSBR gel was observed inside the bamboo fiber. The bamboo fiber treated with NaOH solution in Figure 5e required rinsing with clean water; therefore, the coating material was not found. In Figure 5f, vegetable oil not only coats the surface of the bamboo fiber but also completely penetrates into its interior.

**Figure 6:**
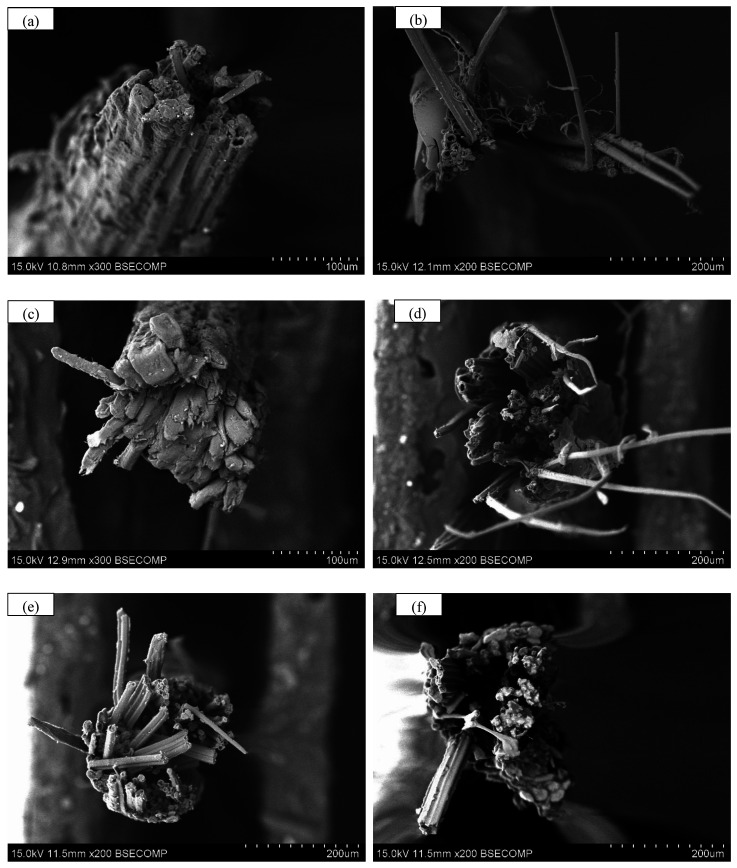
Tensile fracture surfaces of five types of bamboo fibers. (**a**) raw bamboo fibers; (**b**) sodium silicate solution (modulus = 2.3)-treated bamboo fibers; (**c**) sodium silicate solution (modulus = 3.3)-treated bamboo fibers; (**d**) XSBR-treated bamboo fibers; (**e**) NaOH solution-treated bamboo fibers, and (**f**) vegetable oil-treated bamboo fibers.

Figure 6 presents the tensile fracture surfaces of five types of bamboo fibers. All five types of bamboo fibers exhibit brittle fracture characteristics on their tensile fracture surfaces, which is consistent with the tensile behavior observed in Figure 2. Figure 6a displays a relatively smooth fracture surface without an evident necking phenomenon. Figure 6b–f shows tensile fracture surfaces with different lengths of fiber bundle and presents an umbrella-shaped explosive morphology.

## 5. Discussion

As shown in Table 3, the raw bamboo fibers exhibit high tensile strength (with an average value of 587 MPa) and Young’s modulus (with an average value of 23.74 GPa) before water immersion, but both indices decrease significantly after water immersion, with reductions of 60.0% and 28.6%, respectively. It indicates that raw bamboo fibers are highly sensitive to moisture, and their mechanical performance is strongly degraded due to water absorption. At the same time, the strength and stiffness of bamboo fibers treated by different treatment methods also exhibit varying degrees of reduction after water immersion. However, sodium silicate solution treatments, particularly with a modulus of 3.3, lead to the smallest reduction in tensile strength after water immersion compared to other methods. Moreover, sodium silicate solution treatments with a modulus of 2.3 are the only ones that maintain, and even slightly increase, Young’s modulus of bamboo fibers after water immersion. These results indicate that sodium silicate solution treatments are more effective in preserving the mechanical characteristics of bamboo fibers after water immersion.

Macroscopic mechanical behaviors are closely related to the chemical composition and microstructure. FTIR results in Figure 3 show that all treatments alter the functional groups associated with cellulose, hemicellulose, and lignin of bamboo fibers. The disappearance of the peak around 1719.8 cm^−1^ indicates substantial degradation of hemicellulose after all treatments, and the peaks in the 2990–3660 cm^−1^ region correspond to the stretching and vibration of hydrophilic groups (-OH). Sodium silicate solution (modulus = 2.3) and NaOH solution, with stronger alkalinity, cause a significant decrease in -OH-related peaks. In contrast, sodium silicate solution (modulus = 3.3) and XSBR have only a slight effect on the -OH groups and show limited degradation of cellulose and lignin. In addition, the Si–O–related peaks at 1057.2 cm^−1^ and 447.9 cm^−1^ indicate the formation of Si–O–Si hydrophobic networks during thermal curing, confirming that the solidified sodium silicate layer on the bamboo fiber surface possesses a certain degree of hydrophobicity.

As shown in Figure 4 and Figure 5, the surface of the bamboo fibers treated by sodium silicate solution is coated with a solidified sodium silicate layer. Similarly, the surface of the bamboo fibers treated with XSBR is coated with an XSBR gel layer. Both external coating layers provide effective waterproofing performance to bamboo fibers; therefore, softening of the bamboo fibers due to excessive water absorption is avoided. As a result, the original tensile strength and Young’s modulus of the bamboo fibers after immersion are largely preserved. The presence of the coating layers accounts for the seemingly paradoxical experimental phenomenon observed in Section 4.2. Although the sodium silicate solution (modulus = 3.3) treatment does not significantly reduce the number of hydrophilic groups on the bamboo fiber surface, the waterproofing effect of the external coatings still results in superior mechanical performance after water immersion.

As shown in Figure 6, the tensile fracture surface of the treated bamboo fibers shows different lengths of fiber bundle and presents an umbrella-shaped explosive morphology, while such features are not found in the untreated bamboo fibers. The differences in these characteristics can be attributed to structural heterogeneity intensified by the modification treatments. After surface modification with sodium silicate solution, NaOH solution, or vegetable oil, the tensile strength of the bamboo fiber surface is lower than that of the inner bamboo fibers, thereby resulting in a mechanical gradient between the surface and the interior. In contrast, XSBR treatment is distinctive: since the XSBR gel layer is an elastomeric material that bears the load together with the bamboo fibers during the tensile test. Consequently, XSBR-treated bamboo fibers form a hierarchical mechanical gradient structure consisting of “XSBR gel layer—surface-modified bamboo fiber—inner bamboo fiber.”

For the bamboo fibers treated, the inner fibers exhibit higher strength compared with both the surface-modified layer and the XSBR gel layer. When the tensile stress of the bamboo fiber meets a critical value, the surface-modified layer or the XSBR gel layer fractures first. The fracture failure causes an abrupt transfer of tensile stress to the inner bamboo fiber and subsequent stress release, which leads to umbrella-shaped explosive morphology upon ultimate failure of the inner bamboo fiber. For the inner bamboo fiber, weaker fiber bundles fracture earlier than stronger ones, resulting in different lengths of fiber bundles on the fracture surface. Meanwhile, the stress release also shortens the fracture failure process of the inner bamboo fiber, which could explain why the treated bamboo fibers show lower strain values than raw bamboo fibers in Figure 2.

## 6. Conclusions

This study proposed a superior bamboo fiber treatment method that balances environmental friendliness, cost, and mechanical performance, and conducts a comparative analysis based on results from direct tensile tests, SEM tests, and FTIR tests.

1. Direct tensile tests indicate that XSBR-treated bamboo fibers exhibit the most favorable mechanical properties before water immersion, compared with raw bamboo fibers, with a 13.3% and 21.7% increment in tensile strength and Young’s modulus, respectively. After water immersion, mechanical indicators of raw bamboo fibers before water immersion are adopted as a benchmark; all treatment methods result in reduced tensile strength due to water absorption. Bamboo fibers treated with sodium silicate solution (modulus = 3.3) show the smallest reduction in tensile strength, with a value of 36.8%. For Young’s modulus, only the sodium silicate solution (modulus = 2.3) treatment maintains an increment of 4.5%, while all other methods result in a decrement. From the perspective of cost, environmental performance, and mechanical properties, sodium silicate solution is the most promising treatment method for bamboo fibers.
2. Among the treatment methods, sodium silicate solution forms Si-O bonds during thermal curing, resulting in a densely crosslinked Si-O-Si solidified layer on the bamboo fiber surface. Based on the results of the tensile strength reduction rate, the solidified sodium silicate layer exhibits superior waterproofing performance compared to the XSBR gel layer.
3. The tensile fracture surface of raw bamboo fibers is relatively smooth without an evident necking phenomenon. In contrast, the tensile fracture surface of the treated bamboo fibers shows different lengths of fiber bundles and presents an umbrella-shaped explosive morphology. Mechanically, this is reflected in lower strain values of the treated bamboo fibers compared to the raw bamboo fibers.
4. In practical engineering applications, bamboo fiber exhibits considerable potential as a sustainable construction material, with applications including soil reinforcement to improve shear strength, concrete toughening additives, and reinforcement in geopolymer composites. However, the application of bamboo fiber is consistently hindered by high water absorption capacity, which leads to bamboo fiber softening and then significantly affects the mechanical performance and long-term durability of composite materials. This study offers a feasible solution for the application of bamboo fibers in construction engineering.

## Figures and Tables

**Table 1:** Basic chemical composition of bamboo fibers.

Composition	Cellulose (%)	Hemicellulose (%)	Lignin (%)	Acetil Groups (%)	Other Composition (%)
Values	45.5–51.7	20.6–24.1	25.8–29.3	2.1–3.5	<1.0

**Table 2:** Numbers of raw bamboo fibers and four types of treated bamboo fibers, categorized by test conditions (BI: before water immersion, AI: after water immersion).

	Raw Bamboo Fiber	NaOH Solution	Vegetable Oil	XSBR	Sodium Silicate (Modulus = 2.3)	Sodium Silicate (Modulus = 3.3)
BI	10	10	10	10	10	10
AI	10	10	10	10	10	10
total number	120					

**Table 3:** Tensile test results of five types of bamboo fibers before and after water immersion. (BI: before water immersion, AI: after water immersion).

Treatment Method	State	Tensile stress (MPa)	Strain (%)	Young’s Modulus (GPa)	Tensile Strength Reduction Rate (%)	Young’s Modulus
						Reduction Rate (%)
Raw bamboo	BI	587 ± 35.46	2.47 ± 0.66	23.74 ± 6.51	0	0
	AI	235 ± 49.74	1.39 ± 0.32	16.95 ± 3.09	60	28.6
Sodium silicate solution (2.3)	BI	511 ± 75.39	1.97 ± 0.40	25.94 ± 10.11	12.95	−9.27
	AI	358 ± 84.57	1.43 ± 0.51	25.02 ± 6.51	39.01	−4.51
Sodium silicate solution (3.3)	BI	517 ± 44.70	1.90 ± 0.24	27.17 ± 4.27	11.93	−14.45
	AI	371 ± 24.65	1.75 ± 0.30	21.19 ± 3.90	36.8	10.74
NaOH solution	BI	433 ± 34.08	1.70 ± 0.28	25.31 ± 6.65	26.24	−6.61
	AI	288 ± 23.05	1.46 ± 0.11	19.70 ± 3.17	50.94	17.02
XSBR	BI	665 ± 79.65	2.30 ± 0.07	28.90 ± 5.57	−13.29	−21.74
	AI	305 ± 32.48	1.54 ± 0.13	19.85 ± 2.69	48.04	16.39
Vegetable oil	BI	248 ± 32.58	1.15 ± 0.29	21.59 ± 2.03	57.75	9.06
	AI	280 ± 76.49	1.43 ± 0.36	19.58 ± 5.06	52.3	17.52

Note: In the table, positive values (+) indicate that the tensile strength and Young’s modulus of the treated bamboo fibers decreased compared to the values of raw bamboo fibers before water immersion, whereas negative values (−) indicate that the tensile strength and Young’s modulus of the treated bamboo fibers increased compared to the values of raw bamboo fibers before water immersion.

**Table 4:** Assessment of treatment methods.

Treatment Method	Environmental Friendliness	Cost	Tensile Strength Before Water Immersion	Tensile Strength After Water Immersion
Sodium silicate solution (2.3)	favorable	low	relatively high	relatively high
Sodium silicate solution (3.3)	favorable	low	relatively high	the highest
NaOH solution	moderate	low	relatively low	relatively low
XSBR	unfavorable	high	the highest	relatively low
Vegetable oil	favorable	moderate	the lowest	the lowest

## Data Availability

The original contributions presented in this study are included in the article. Further inquiries can be directed to the corresponding author.
